# Presenilin-Deficient Neurons and Astrocytes Display Normal Mitochondrial Phenotypes

**DOI:** 10.3389/fnins.2020.586108

**Published:** 2021-01-22

**Authors:** Sabrina Contino, Nuria Suelves, Céline Vrancx, Devkee M. Vadukul, Valery L. Payen, Serena Stanga, Luc Bertrand, Pascal Kienlen-Campard

**Affiliations:** ^1^Alzheimer Research Group, Molecular and Cellular Division (CEMO), Institute of Neuroscience, Université Catholique de Louvain, Brussels, Belgium; ^2^Laboratory of Advanced Drug Delivery and Biomaterial (ADDB), Louvain Drug Research Institute (LDRI), Université Catholique de Louvain, Brussels, Belgium; ^3^Neuroscience Institute Cavalieri Ottolenghi, Department of Neuroscience, University of Torino, Torino, Italy; ^4^Pole of Cardiovascular Research, Institute of Experimental and Clinical Research, Université Catholique de Louvain, Brussels, Belgium

**Keywords:** presenilins, OXPHOS, mitochondria, astrocyte, neuron, Alzheimer's disease

## Abstract

Presenilin 1 (PS1) and Presenilin 2 (PS2) are predominantly known as the catalytic subunits of the γ-secretase complex that generates the amyloid-β (Aβ) peptide, the major constituent of the senile plaques found in the brain of Alzheimer's disease (AD) patients. Apart from their role in γ-secretase activity, a growing number of cellular functions have been recently attributed to PSs. Notably, PSs were found to be enriched in mitochondria-associated membranes (MAMs) where mitochondria and endoplasmic reticulum (ER) interact. PS2 was more specifically reported to regulate calcium shuttling between these two organelles by controlling the formation of functional MAMs. We have previously demonstrated in mouse embryonic fibroblasts (MEF) an altered mitochondrial morphology along with reduced mitochondrial respiration and increased glycolysis in PS2-deficient cells (PS2KO). This phenotype was restored by the stable re-expression of human PS2. Still, all these results were obtained in immortalized cells, and one bottom-line question is to know whether these observations hold true in central nervous system (CNS) cells. To that end, we carried out primary cultures of PS1 knockdown (KD), PS2KO, and PS1KD/PS2KO (PSdKO) neurons and astrocytes. They were obtained from the same litter by crossing PS2 heterozygous; PS1 floxed (PS2^+/−^; PS1^flox/flox^) animals. Genetic downregulation of PS1 was achieved by lentiviral expression of the Cre recombinase in primary cultures. Strikingly, we did not observe any mitochondrial phenotype in PS1KD, PS2KO, or PSdKO primary cultures in basal conditions. Mitochondrial respiration and membrane potential were similar in all models, as were the glycolytic flux and NAD^+^/NADH ratio. Likewise, mitochondrial morphology and content was unaltered by PS expression. We further investigated the differences between results we obtained here in primary nerve cells and those previously reported in MEF cell lines by analyzing PS2KO primary fibroblasts. We found no mitochondrial dysfunction in this model, in line with observations in PS2KO primary neurons and astrocytes. Together, our results indicate that the mitochondrial phenotype observed in immortalized PS2-deficient cell lines cannot be extrapolated to primary neurons, astrocytes, and even to primary fibroblasts. The PS-dependent mitochondrial phenotype reported so far might therefore be the consequence of a cell immortalization process and should be critically reconsidered regarding its relevance to AD.

## Introduction

Alzheimer's disease (AD) is the most prevailing age-related neurodegenerative disease. Its cost and impending rise owed to societal aging makes it a major social concern and a critical public health burden. This pathology is characterized by the progressive spreading of two typical lesions in the brain: senile plaques and neurofibrillary tangles (NFTs), that are extracellular deposits of the amyloid-β peptide (Aβ) and intracellular aggregates of hyperphosphorylated tau protein, respectively (Serrano-Pozo et al., 2011). The most admitted comprehensive hypothesis for the onset and development of AD is the amyloid cascade hypothesis (ACH) (Hardy, 2006). It postulates that changes in Aβ production, accumulation, or clearance are triggering events that induce the formation of NFTs eventually leading to neurodegeneration and clinical symptoms. Many efforts have been undertaken to better understand the pathological processes responsible for the altered production of Aβ. They led to the identification of the Amyloid Precursor Protein (APP), and the Presenilin proteins (PS1 and PS2) that are the catalytic subunits of the γ-secretase that releases Aβ upon amyloidogenic processing of APP (Goate et al., 1991; Rogaev et al., 1995; Sherrington et al., 1995). However, clinical trials targeting the amyloid pathology have failed so far to improve cognitive deficits, even though they reduce the amyloid load under certain conditions (Ceyzeriat et al., 2020). Amyloid deposition appears to take place very early in pre-clinical stages and might be associated to brain dysfunctions that appear prior to clinical symptoms. Metabolic aspects of the pathology are widely studied in that context. For instance, the ^18^F-FDG-PET scan is an established imaging standard for neuronal dysfunction in the diagnostic workup of AD-patients (Mosconi et al., 2010; Herholz, 2012; Shivamurthy et al., 2015). It was used to evidence that hypometabolism and brain atrophy appear before clinical symptoms in patients.

Mitochondria play a central role in cellular metabolism, and mitochondrial function is known to be particularly affected in the disease (Garcia-Escudero et al., 2013). Several studies have put forth a “mitochondrial cascade hypothesis” (Swerdlow and Khan, 2004; Stanga et al., 2020), according to which mitochondrial alterations are capable of initiating compensatory events that would result in the histopathological sequence of AD, including an increased production of the amyloid-β (Aβ) peptide, and thus they can be considered as an upstream event in the development of the pathology. Alternatively, mitochondrial dysfunction has also rather been suggested as a consequence of pathological processes such as amyloid deposition (Pagani and Eckert, 2011; Swerdlow et al., 2014; Swerdlow, 2018). Despite this controversy, there is little doubt that mitochondrial dysfunction contributes to AD pathogenesis, as evidenced in animal models of AD (Yao et al., 2009; Dixit et al., 2017; Long and Holtzman, 2019) and samples from AD patients (Wang et al., 2009; Martin-Maestro et al., 2017; Adav et al., 2019). Major consequences of mitochondrial dysfunction such as increased Reactive Oxygen Species (ROS) production (Dixit et al., 2017), impaired balance of fusion/fission with altered morphology (Wang et al., 2009), decreased oxidative capacity and decreased motility (Correia et al., 2016) are described in AD pathogenesis.

Mitochondrial alterations found in AD have been associated to functional changes of the major AD proteins, namely APP, Tau, and PSs (Garcia-Escudero et al., 2013). Only Presenilin 1 and 2 (PS1 and PS2) were yet clearly shown to be involved in mitochondrial function. PS1 and PS2 are encoded by two homologous genes, *PSEN1* and *PSEN2*, respectively. Mutations in *PSEN* genes are responsible for the majority of inherited AD cases (Hardy, 2006). Apart from their involvement in Aβ production (as the catalytic subunits of the γ-secretase), functions attributed to PSs can be divided into two categories: γ-secretase-dependent or γ-secretase-independent (Vetrivel et al., 2006; Zhang et al., 2013). The γ-secretase independent functions of PSs are known to be involved in synaptic transmission, endosome-lysosome trafficking, Wnt signaling, and calcium homeostasis. The involvement of PSs in calcium homeostasis has been particularly investigated. PSs could interact with Inositol trisphosphate receptor (IP3R) (Cheung et al., 2008), sarco-/endoplasmic reticulum (ER) Ca^2+^ ATPase (SERCA), or Ryanodine receptors (RyR) (Green et al., 2008; Wu et al., 2013) to regulate intracellular calcium signaling. PSs were also suggested to directly act as a low-conductance, passive ER Ca^2+^ channel (Nelson et al., 2007). Finally, PSs could also regulate the calcium crosstalk between the mitochondria and the ER by regulating their apposition through a particular domain called mitochondria-associated membranes (MAMs) (Filadi et al., 2016). Indeed, studies have shown that PSs are enriched in MAMs, which are lipid-raft-like structures. MAMs are considered as functional compartments (Area-Gomez et al., 2009) due to their implication in cellular pathways such as inflammation, mitophagy, and lipid production (Filadi et al., 2017). PS2 was found to physically interact with Mitofusin 2 to regulate MAMs organization and calcium shuttling in mouse embryonic fibroblasts (MEF) (Filadi et al., 2016). Disruption of this interaction and of the consecutive calcium crosstalk was reported in the SH-SY5Y cell line transfected with siRNA targeting PS2 (Zampese et al., 2011). Importantly, impaired calcium influx from the ER to mitochondria is implicated in the regulation of the oxidative phosphorylation (OXPHOS) and can lead to mitochondrial defects. Using MEF cell lines, we observed an altered mitochondrial phenotype related to the absence of PS2 and not PS1 (Contino et al., 2017). PS2 deficiency results in defective mitochondrial cristae correlated to an impaired OXPHOS capacity and a modified redox state (NAD^+^/NADH ratio). This was compensated by an increased glycolytic capacity, sustaining a stable ADP/ATP ratio. Together with other studies (Wang et al., 2009; Pagani and Eckert, 2011; Correia et al., 2016; Dixit et al., 2017), this led to the hypothesis that PS-dependent mitochondrial dysfunction could represent a major pathway in AD pathogenesis.

In this context, we investigated for the first time mitochondrial activity in primary cultures of neurons and astrocytes, which are more relevant to AD than immortalized cells. We performed primary cultures of cells expressing both PSs (wild-type, WT), one of them (PS single-knockdown/knockout, referred to as PS1KD and PS2KO), or none (combined PS1KD-PS2KO, referred to as PSdKO). Our aim was to investigate (i) if mitochondrial-related deficits appear in the absence of PSs in these cells and, if so, (ii) to identify which PS is involved in this phenotype and the possible mechanisms underpinning mitochondrial dysfunction in neurons and/or astrocytes. Surprisingly, we did not find any metabolic deficit in the different PS knockdown/knockout primary nerve cells, even in the absence of both PSs (PSdKO). Likewise, mitochondrial morphology and content were not altered in PS2KO primary neurons, contrary to what was observed in PS2-deficient MEF cell lines (Contino et al., 2017). We further studied the mitochondrial activity in PS2KO primary MEF and did not observe any mitochondrial defect. The obtained evidence leads us to conclude that PS-dependent mitochondrial alterations observed in immortalized cells may provide information about PS-dependent cancer processes, but give no straightforward indication about mitochondrial dysfunction in AD.

## Materials and Methods

### Animal Models

Presenilin 2 knockout (KO) (#005617) (Herreman et al., 1999) and Presenilin 1 floxed (#004825) (Yu et al., 2000) mice, both in a C57BL/6 background, were obtained from Jackson Laboratories (Bar Harbor, USA). All animal procedures and experiments were approved and performed in agreement with the UCLouvain animal care committee's regulations (code number 2016/UCL/MD/016). Animals were housed on a 12 h light/dark cycle in a standard animal care facility with access to water and food *ad libitum*.

For primary cell cultures, generation of the different genotypes in the same litter was obtained from crossing PS2 heterozygous and PS1 floxed (PS2^+/−^; PS1^flox/flox^) animals followed or not by viral transduction. Indeed, *PSEN1* gene deletion was achieved by viral transduction of Cre recombinase in floxed PS1 primary cultures. Lentivirus expressing GFP (mock control) or CRE-GFP were used for transduction at DIV1 (one day of *in vitro* culture) for neurons and DIV17 for astrocytes. Following genotyping and infection we could obtain in the same litter: control non-infected **Ct** (PS2^+/+^; PS1^flox/flox^, non-infected), **PS2KO** (PS2^−/−^; PS1^flox/flox^, non-infected); control infected named **Mock** (PS2^+/+^; PS1^flox/flox^, infected with GFP); **PS1KD** (PS2^+/+^; PS1^flox/flox^, infected with CRE-GFP); and **PSdKO** (PS2^−/−^; PS1^flox/flox^, infected with CRE-GFP).

### Primary Neuronal Cultures

Primary cultures of neurons were performed as previously described (Opsomer et al., 2020) on E17 mouse embryos. Cortices and hippocampi were isolated by dissection on ice cold HBSS (Thermo Fisher Scientific, Waltham, USA) and meninges were removed. Tissues were then dissociated by pipetting up and down 15 times with a glass pipette in HBSS glucose 5 mM medium. Dissociation was repeated 10 times with a flame-narrowed glass pipette. After sedimentation for 5 min, the supernatant containing the neurons was settled on a bed of 4 ml of Fetal Bovine Serum (FBS) and centrifuged at 1,000 × *g* for 10 min. The pellet was resuspended in Neurobasal^®^ medium enriched with 1 mM L-glutamine, 5 mM glucose, and 2% v/v B-27^®^ supplement medium. Cells were plated at 200,000 cells/cm^2^ on pre-coated poly-L-lysine dishes and cultured (37°C, 5% CO_2_ and humidified atmosphere). Half media changes were performed every 2 days and neurons were cultured for 11 days (DIV11) before being utilized for experiments. To get PS1KD and PSdKO, neurons were infected at DIV1 and all media were changed at DIV2.

### Primary Astrocyte Cultures

For primary astrocyte cultures, the brains of mouse pups aged 2 days were dissected to isolate cortices on ice cold HBSS. Tissues were triturated 15 times with a glass pipette and 10 times with a flame-narrowed glass pipette. Tubes were centrifuged at 1,000 × *g* for 5 min. Pellets were resuspended in HBSS and centrifuged at 1,700 × *g* for 20 min on a 30% Percoll gradient. Astrocytes were collected from the interphase, washed in 10 ml of HBSS, and centrifuged for 5 min at 1,500 × *g*. Pellets were resuspended and plated in DMEM-glutaMAX medium (Thermo Fisher Scientific, Waltham, USA) supplemented with 10% FBS (Biowest, Nuaillé, France), 50 mg/ml penicillin–streptomycin, and 50 mg/ml fungizone. Cells were left to proliferate in flasks for 15 days at 37°C and 5% CO_2_, and media were changed every 4–5 days. After 15 days, astrocytes were plated and further cultured in DMEM-glutaMAX with 10% FBS. Two days later, transduction with lentivirus was achieved when necessary and differentiation was induced by reducing the concentration of FBS to 3% for 7 days before performing experiments.

### Primary and Immortalized Mouse Embryonic Fibroblasts (MEF)

Immortalized MEF were cultured as previously described (Stanga et al., 2018). Primary cultures of MEF were performed on E16 embryos. Chest skin was isolated on ice and then grinded into pieces with a blade. These pieces were dissociated and incubated twice at 37°C for 10 min in trypsin (Life Technologies, Carlsbad, USA). DMEM low glucose (5.5 mM) (Sigma-Aldrich, St Louis, USA) enriched with 10% FBS and 1% pen/strep was added for an incubation of 5 min at room temperature (RT). Supernatants were collected and centrifuged at 1,000 × *g* for 5 min at RT. Pellets were resuspended in 10 ml of DMEM medium and plated in petri dishes. Once at confluence, cells were plated for experiments.

### Lentiviral Particles

Lentiviral particles expressing CRE recombinase were used to delete floxed *PSEN1*. Plasmids pCMV-GFP (Mock) and pCMV-CRE-GFP for lentiviral production were purchased from Cellomics Technology (Halethorpe, USA). Amplification and purification of the different plasmids were performed using the Plasmid Midi kit (Qiagen, Hilden, Germany). Lentiviral production was carried out by transfecting HEK293-T cells in 10 cm dishes (2 × 10^6^ cells/dish) with CRE-GFP or GFP vectors, pMD2.G (Addgene#12259), and pCMV-dR8.2 (Addgene#12263). At 48 h after transfection, cells were harvested by flushing with the medium and centrifuged at 1,500 × *g* for 10 min at 4°C. The supernatant was filtered with Acrodisc^®^ 0.45 μm filters (Pall, NYC, USA). Then, 1/3 (v/v) of LentiX™ Concentrator reagent (Clontech, Mountain View, USA) was added and incubated overnight (o/n). After centrifugation at 1,500 × *g* for 45 min at 4°C, the pellet was resuspended in 20 μl per dish of DMEM without serum and stored at −80°C; 10 μl of concentrated virus was used to infect 1,600,000 neurons or 300,000 astrocytes.

### Western Blotting (WB)

WB was performed on cell lysates obtained by harvesting cells with lysis buffer (Tris 125 mM pH 6.8, 4% sodium dodecyl sulfate, 20% glycerol) with Complete Protease Inhibitor Cocktail (Roche, Basel, Switzerland) and sonicating, as previously described (Stanga et al., 2016). Protein concentration was determined using the BCA Protein assay kit (Pierce, Rockford, USA). Then, 15 μg of total proteins diluted in lysis buffer with NuPAGE^®^ LDS Sample Buffer and 50 mM DTT was heated at 70°C (except for the detection of OXPHOS subunits, heated at 37°C). Samples were loaded and separated onto NuPAGE^®^ 4–12% Bis-Tris gel (Life Technologies, Carlsbad, USA) with a NuPAGE^®^ MES SDS running buffer (Life Technologies, Carlsbad, USA). Proteins were then transferred onto an Amersham™ nitrocellulose membrane (Little Chalfont, UK) with NuPAGE^®^ transfer buffer (Life Technologies, Carlsbad, USA) for 2 h at 30 V. After blocking with non-fat dry milk (Darmstadt, Germany) (5% in PBS and 0.05% Tween-20) for a minimum of 30 min, the membrane was incubated o/n with the primary antibody diluted in PBS 0.05% Tween. Secondary antibody coupled to horse radish peroxidase was incubated 1 h at RT. Membranes were detected using ECL PerkinElmer^®^ (Waltham, USA). ImageJ software was used for densitometric analysis of the different immunoreactive bands.

Antibodies used and their dilutions are the following: Anti-Actin (1:3,000; Abcam, Cambridge, United Kingdom); Anti-APP C-ter (1:2,500; generous gift of N. Sergeant, INSERM U422, Lille, France); Anti-DRP1 (1:1,000; Cell Signaling, Danvers, USA); Anti-mouse (1:10,000; GE Healthcare, Little Chalfont, United Kingdom); Anti-OPA1 (1:1,000; Cell Signaling, Danvers, USA); Anti-TIM23 (1:500; Santa Cruz, California, USA); Anti-OXPHOS Cocktail (1:1,000; Abcam, Cambridge, United Kingdom); Anti-Presenilin 1 (1:1,000; Cell Signaling, Danvers, USA); Anti-Presenilin 2 (1:1,000; Cell Signaling, Danvers, USA); Anti-rabbit (1:10,000; GE Healthcare, Little Chalfont, United Kingdom); Anti-TOM20 (1:1,000; Proteintech, Rosemont, USA); Anti-Tubulin (1:3,000, Abcam, Cambridge, United Kingdom).

### Immunocytochemistry (ICC)

ICC were performed as previously described (Hage et al., 2014). Cells were seeded on pre-coated poly-L-lysine coverslips in 24 well plates at the density of 200,000/well for neurons or 50,000/well for astrocytes. Cells were fixed with PBS/paraformaldehyde 4% for 10 min and rinsed three times for 5 min with PBS. Cells were then permeabilized with a solution of PBS/Triton 0.3% for 30 min and non-specific sites were blocked with PBS/Triton 0.3%/FBS 5% for 30 min. Primary antibodies diluted in the blocking solution were incubated o/n at 4°C. After 3 washes of 10 min with PBS, cells were incubated with DAPI (1:2,000; Sigma-Aldrich, St Louis, USA) and secondary antibodies diluted in the blocking solution. Dilutions of the antibodies were as follows: chicken anti-glial-fibrillary acidic protein (GFAP2; 1:1,1000; Abcam, Cambridge, United Kingdom); mouse anti-microtubule associated protein (MAP2; 1:1,000; Sigma-Aldrich, St Louis, USA); rabbit anti-TOM20 (1:500, Proteintech, Rosemont, USA); Alexa 488 anti-rabbit (1:500; Life Technologies, Carlsbad, USA); Alexa 568 anti-mouse (1:500; Life Technologies, Carlsbad, USA); Alexa 647 anti-chicken (1:500; Life Technologies, Carlsbad, USA).

To characterize neuronal and astrocyte general morphology, images from MAP2 and GFAP stained-cells were acquired on EVOS FL Auto microscope (Invitrogen) with RFP (Alexa Fluor 554), and CY5 (Alexa Fluor 647) EVOS LED light cubes and analyzed with ImageJ software.

To evaluate mitochondrial morphology after TOM20 staining, samples were examined by confocal microscopy using a confocal server spinning disc Zeiss platform equipped with a ×100 objective. For each image, z-stacks were taken from the entire three-dimensional structure and the maximum intensity projection was obtained in ImageJ.

### *In silico* Analysis of Mitochondrial Morphology

Morphological analysis of mitochondria was performed *in silico* using a plug-in of ImageJ, the toolset MiNA (Mitochondrial Network Analysis) (Valente et al., 2017). MiNA allows semi-automated analysis and consists in images' preprocessing, to ensure quality, conversion to binary image, and in the production of the final skeleton for the quantitative analysis, as previously described (Calabrese et al., 2020). Briefly, images were opened on ImageJ and processed as follows: 1-Process/Filters/UnsharpMask; 2-Process/EnhanceLocal Contrast (CLAHE); 3-Process/Filters/Median; 4-Process/Binary/MakeBinary; 5-Process/Binary/Skeletonize; 6-Analyze/Skeleton/AnalyzeSkeleton(2D/3D); 7-Plugins/StuartLab/MiNAScripts/MiNAAnalyzeMorphology.

### Mitochondrial Membrane Potential (ΔΨ)

Fluorescent cationic probe tetramethylrhodamine methyl ester (TMRM) (Sigma-Aldrich, St. Louis, USA) was used to evaluate the ΔΨ. As a control, we used the uncoupling agent Carbonyl cyanide-4-(trifluoromethoxy) phenylhydrazone (FCCP) (Sigma-Aldrich, St. Louis, USA). Cells were plated in 96-well plates at a density of 60,000/well for neurons or 15,000/well for astrocytes. Cells were incubated for 30 min at 37°C with TMRM (30 nM) with or without FCCP (10 μM) diluted in KREBS medium. Cells were then washed with KREBS solution and fluorescence was read with the plate reader VICTOR^®^ Multilabel Plate Reader (PerkinElmer). Data were normalized to the total amount of protein measured by the Bradford assay kit (Bio-Rad Laboratories, California, USA).

### Mitochondrial Oxygen Consumption

Oxygen consumption rate (OCR) was measured with the Seahorse XF96 bioenergetic analyzer (Seahorse Bioscience; Massachusetts, USA). Cells were seeded in a Seahorse 96-well plate at different densities (60,000/well for neurons, 15,000/well for astrocytes, or 20,000/well for MEF). To analyze the effect of the inhibition of γ-secretase activity on OCR, cells were treated with N-[N-(3,5-difluorophenacetyl)-L-alanyl]-sphenylglycine t-butyl ester (DAPT) (Calbiochem, Camarillo, CA, USA) 24 h before performing the experiment. Once differentiation was completed, the medium was exchanged with the conditional medium (culture medium without sodium bicarbonate and FBS) and incubated without CO_2_ at 37°C for 1 h. Inhibitors targeting the different mitochondrial complexes (Cell Mito Stress Test kit, Seahorse Bioscience) were added automatically and sequentially to the cells during the experiment to measure the basal respiration, the coupling, and the spare respiratory capacity. The sequence of the inhibitors used was: Oligomycin (1 μM); FCCP (0.5 μM for neurons and 1 μM for astrocytes and MEF); Rotenone and antimycin A (0.5 μM). Results were normalized to the total amount of protein measured by the Bradford assay kit (Bio-Rad Laboratories, California, USA).

### NAD^+^/NADH Ratio

NAD^+^/NADH ratio was measured with the bioluminescent NAD^+^/NADH-Glo assay kit (Promega, Wisconsin, USA) according the manufacturer's instructions. Cells were seeded in a 96-well plate at different densities (60,000/well for neurons, 10,000/well for astrocytes or 20,000/well for MEF). Once the differentiation was completed, cells were rinsed with PBS and then lysed with the basis solution 1 % dodecyltrimethylammonium bromide (DTAB). Samples were split for a basic or acid treatment and were heated at 60°C for 15 min. The reduced form was decomposed in the acidic solution and the oxidized form was selectively decomposed in the basic solution. After neutralization, samples were mixed with NAD^+^/NADH-Glo^TM^ detection reagent and incubated for 45 min to induce reaction. Luminescence was read on the GloMax^®^ 96-well plate luminometer (Promega, Wisconsin, USA).

### Glycolytic Flux

Glycolytic rate was analyzed by the measurement of the detritiation rate of [3-^3^H] glucose as previously described (Contino et al., 2017). Briefly, cells were seeded in a 12-well plate at density of 800,000/well for neurons, 100,000/well for astrocytes, or 200,000/well for MEF. Tritiated glucose (0.2 μCi/ml; Perkin-Elmer; Massachusetts, USA) was added to the medium (including 5.5 mM glucose) for 30 min. After medium collection, the tritiated water resulting from detritiated glucose was separated from the non-transported tritiated glucose by column chromatography and measured with the Tri Carb 2,810 liquid scintillation analyzer (Perkin Elmer; Massachusetts, USA) as described previously (Marsin et al., 2002). Data were normalized to the total amount of protein measured by BCA assay (Thermo Scientific, Rockford, USA).

### ATP Level

ATP level was measured with the bioluminescent ATPlite^®^ assay kit (Promega, Wisconsin, USA) according the manufacturer's instructions. Cells were seeded in a 96-well plate at a density of 60,000/well for neurons or 10,000/well for astrocytes. On the day of the experiment, medium was replaced by 75 μl of fresh medium for 30 min at room temperature. Then, 75 μl of kit's solution was added and after 10 min of incubation, luminescence was read on the GloMax^®^ 96-well plate luminometer (Promega, Wisconsin, USA).

### RT-qPCR Quantification of Mitochondrial DNA Content

Relative mitochondrial DNA (mtDNA) content was quantified from isolated total DNA as previously described (Missios et al., 2014). Briefly, neurons were digested with proteinase K (100 μg/ml) in lysis buffer [100 mM NaCl, 100 mM Tris–HCl (pH 8.0), 1 mM EDTA, (pH 8.0) and 1% (w/v) SDS] at 50 °C o/n and genomic DNA was purified with phenol/chloroform method. Real-time quantitative polymerase chain reaction (RT-qPCR) was performed using different primer pairs to detect the mitochondrial genes *Cyt-b* and *Nd1*, and the nuclear gene *H19*. The relative mitochondrial DNA content was calculated by the ΔΔCt method. Previously described primers (Ferrara-Romeo et al., 2020) were used as follows: CYTB-F 5′-ATTCCTTCATGTCGGACGAG-3′, CYTB-R 5′-ACTGAGAAGCCCCCTCAAAT-3′, ND1-F 5′-AATCGCCATAGCCTTCCTAACAT-3′, ND1-R 5′-GGCGTCTGCAAATGGTTGTAA-3′, H19-F 5′-GTACCCACCTGTCGTCC-3′, H19-R 5′-GTCCACGAGACCAATGACTG-3′.

### Statistical Analysis

Number of samples are indicated in figure legends with “n =” and number of independent experiments with “N =.” GraphPad Prism software (GraphPad Software, La Jolla, CA, USA) was used to analyze the data and perform the statistical analyses. Normality was assessed with Shapiro Wilk test (GraphPad Prism). Parametric test (Student's *t-*test, ANOVA and Tukey's multiple comparison test) was applied if the data followed normal distribution. Otherwise, non-parametric test (Mann-Whitney test, Kruskal-Wallis and Dunn's multiple comparison test) was used. Significance is indicated as: *p < 0.05, **p < 0.01, ***p < 0.001, ****p < 0.0001.

## Results

### Generation of PS1KD, PS2KO, PSdKO Primary Nerve Cells and Characterization of the Primary Cultures

**Figure 1:**
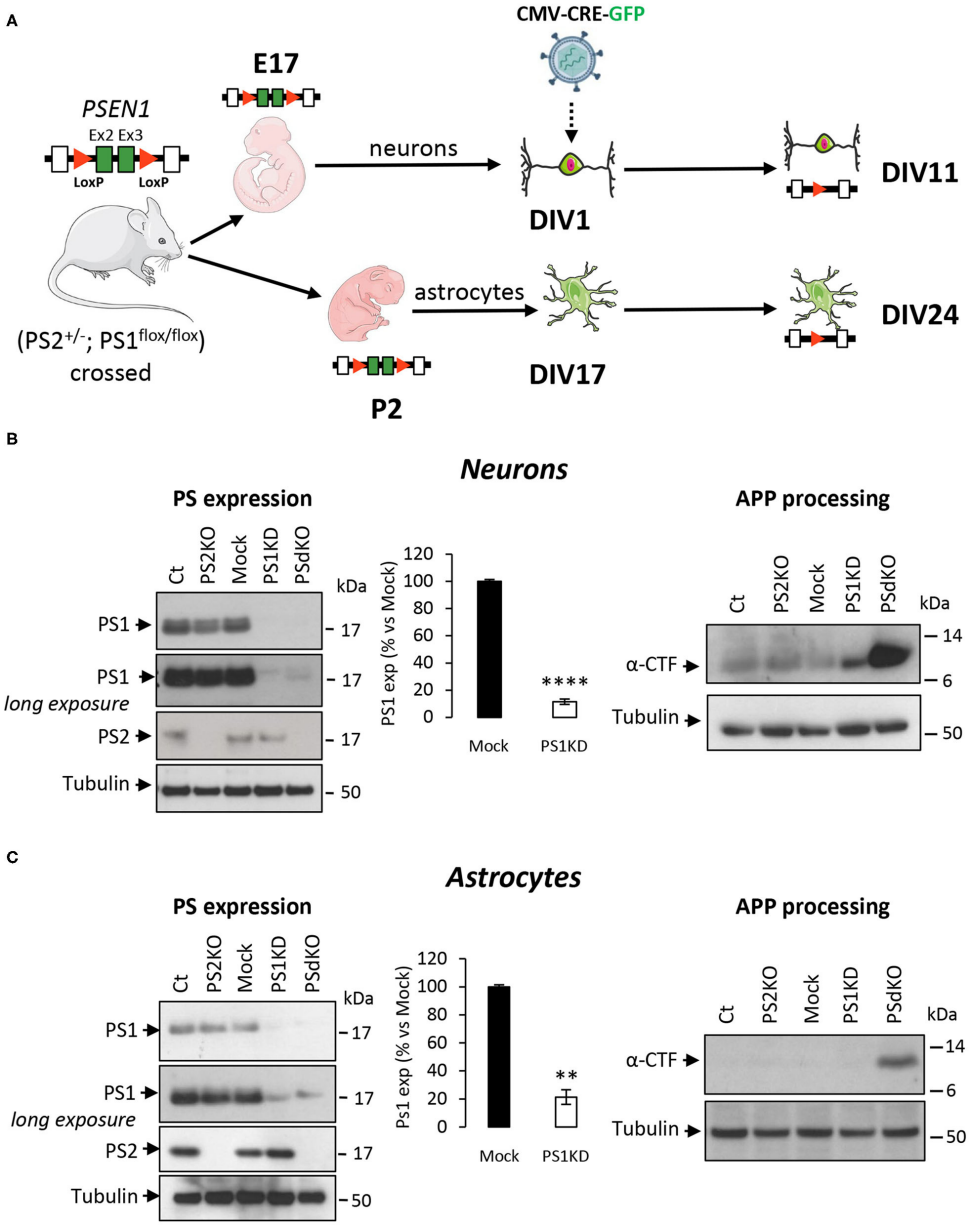
Experimental workflow and model characterization in PS-defective primary nerve cells. **(A)** Workflow description of the experiments with specificity for *PSEN1* deletion. Primary neuronal and astrocyte cultures were performed on embryos at day 17 (E17) and postnatal pups at day 2 after birth (P2), respectively. Generation of the different genotypes in the same litter were obtained from crossing PS2 heterozygous and PS1 floxed (PS2^+/−^; PS1^flox/flox^) animals followed where necessary by viral transduction. At DIV1 for neurons and DIV17 for astrocytes, cells were either non-infected (Ct and PS2KO) or infected with GFP (Mock) or CRE-GFP to induce *PSEN1* gene deletion (PS1KD and PSdKO). Experiments were performed after 11 days for neurons and 24 days (including 7 days of differentiation) for astrocytes. Ex2 and Ex3 = *PSEN1* exons 2 and 3, respectively. **(B,C)** Representative WBs showing PS1 and PS2 expression profiles (left panel) and APP C-terminal fragment (α-CTFs; right panel) in total cell lysates from primary neuronal **(B)** and astrocyte **(C)** cultures. Tubulin was used as loading control. Quantification of PS1 expression (means ± sem) is given as percentage of signal measured in control cells (Mock); ****p < 0.0001; **p < 0.01; Student's *t*-test for neurons results and Mann-Whitney test for astrocytes results (*N* = 6).

**Figure 2:**
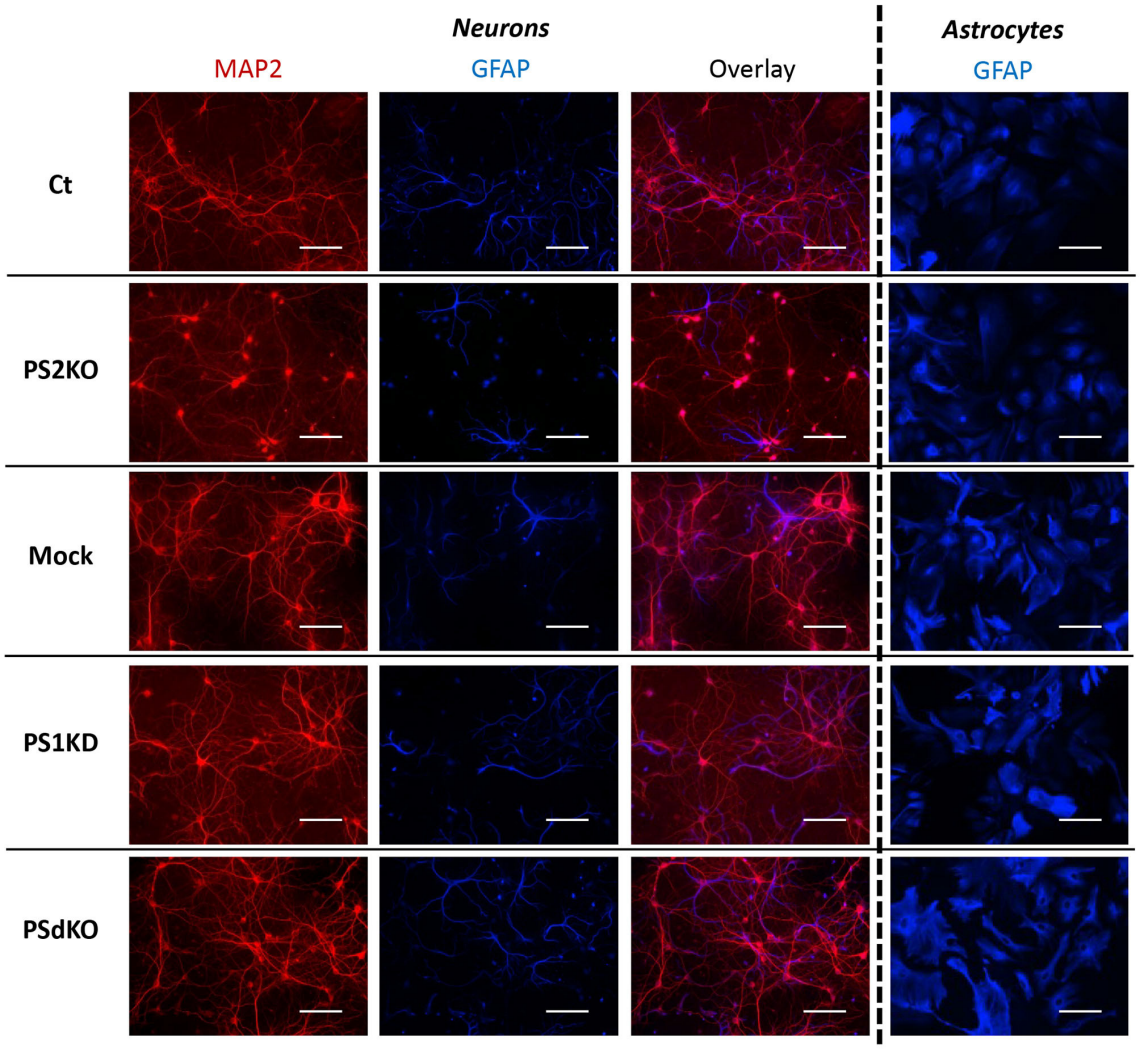
Morphology of PS-defective primary neuronal and astrocyte cultures. On the left side, a primary culture of neurons at DIV11 immuno-stained with glial-specific protein GFAP (blue) and the neuron-specific protein MAP2 (red) antibodies. On the right side, a primary culture of astrocytes at DIV24 immuno-stained with GFAP (blue) antibody. Scale bar = 100 μm.

Metabolic analyses were performed on primary neurons and astrocytes produced at embryonic day 17 (E17) and at postnatal day 2 (P2), respectively. Neurons were cultured for 1 day (DIV1) and astrocytes for 17 days (DIV17) prior to lentiviral transduction and were further maintained in culture until DIV11 for neurons and DIV24 for astrocytes. The experimental workflow is shown in Figure 1A. The different culture times (DIV) were chosen in order to obtain differentiated and active primary neurons and astrocytes (Schildge et al., 2013; Charlesworth et al., 2015). Our previous studies indicated that under these culture conditions, neurons acquire properties of differentiated neurons from DIV7 on (Opsomer et al., 2020) and that the spontaneous calcium oscillation of a functional neuronal network are readily observed at DIV10 (Santos et al., 2009, 2010). Thus, we can reasonably assume that the primary neurons in culture studied here were mature and interconnected in a functional network. Generation of the different genotypes was obtained in the same litter by crossing PS2 heterozygous and PS1 floxed (PS2^+/−^; PS1^flox/flox^) animals, followed when necessary by viral transduction of the Cre recombinase. This approach has been used because although PS2 knockout (PS2^−/−^; PS2KO) cells can be obtained from the viable PS2 full KO mice (Herreman et al., 1999), PS1KO and PSdKO mice present a lethal phenotype at E17 and E12, respectively (Shen et al., 1997; Donoviel et al., 1999). Therefore, to obtain PS1KO and PSdKO primary cultures, *PSEN1* deletion was achieved by viral transduction of Cre recombinase in PS1^flox/flox^ primary cultures (Figure 1A). Lentivirus expressing GFP (mock control) or CRE-GFP were used at DIV1 (primary neurons) or at DIV17 (primary astrocytes). Efficiency of infection was monitored by evaluating GFP fluorescence intensity. Three days after infection, most cells were GFP positive and GFP expression was maintained until the day of the analysis. An example of GFP and CRE-GFP infected neurons is shown in Supplementary Figure 1. We evaluated the expression level of PS1 and PS2 by WB in neuronal and astrocyte cultures (Figure 1B). Cre-mediated excision of *PSEN1* was very efficient under these conditions, with 90% decrease in PS1 expression when compared to mock-infected control (Figure 1B). However, since a residual PS1 expression was still observed after the infection, we consider the obtained cultures as a model of PS1 knockdown (PS1KD). Given that APP is a major substrate of the γ-secretase and critically involved in AD, we evaluated by WB the accumulation of APP C-terminal fragments (α-CTFs and β-CTFs) following PSs deletion. CTFs are the direct substrates of the γ-secretase that accumulate when γ-secretase activity is blocked. An important accumulation of the CTFs (likely α-CTFs) was observed in PSdKO neurons and astrocytes (Figures 1B,C). CTF accumulation is also observed in PS1KD neuronal cultures, in line with the fact that PS1 is more expressed in neurons than astrocytes (Lee et al., 1996; Lah et al., 1997). The accumulation of CTFs is very low in the absence of PS2 in primary neurons. This is in agreement with the fact that PS2 only marginally contributes to APP processing when PS1 is present. To further characterize our model, we checked the overall morphology of the various PS-deficient cells at DIV11 (neurons) and DIV24 (astrocytes) by immunostaining cells with astrocytic (GFAP) and neuronal (MAP2) markers (Figure 2). Protoplasmic astrocytes cultures were pure while some activated astrocytes were present in neuronal cultures (±10%). There were no apparent differences in terms of morphology or growth between Ct, mock, PS1KD, PS2KO, and PSdKO cells.

### Membrane Potential (ΔΨ) and OXPHOS Complexes Expression Are Not Affected in PS-Deficient Primary Nerve Cells

**Figure 3:**
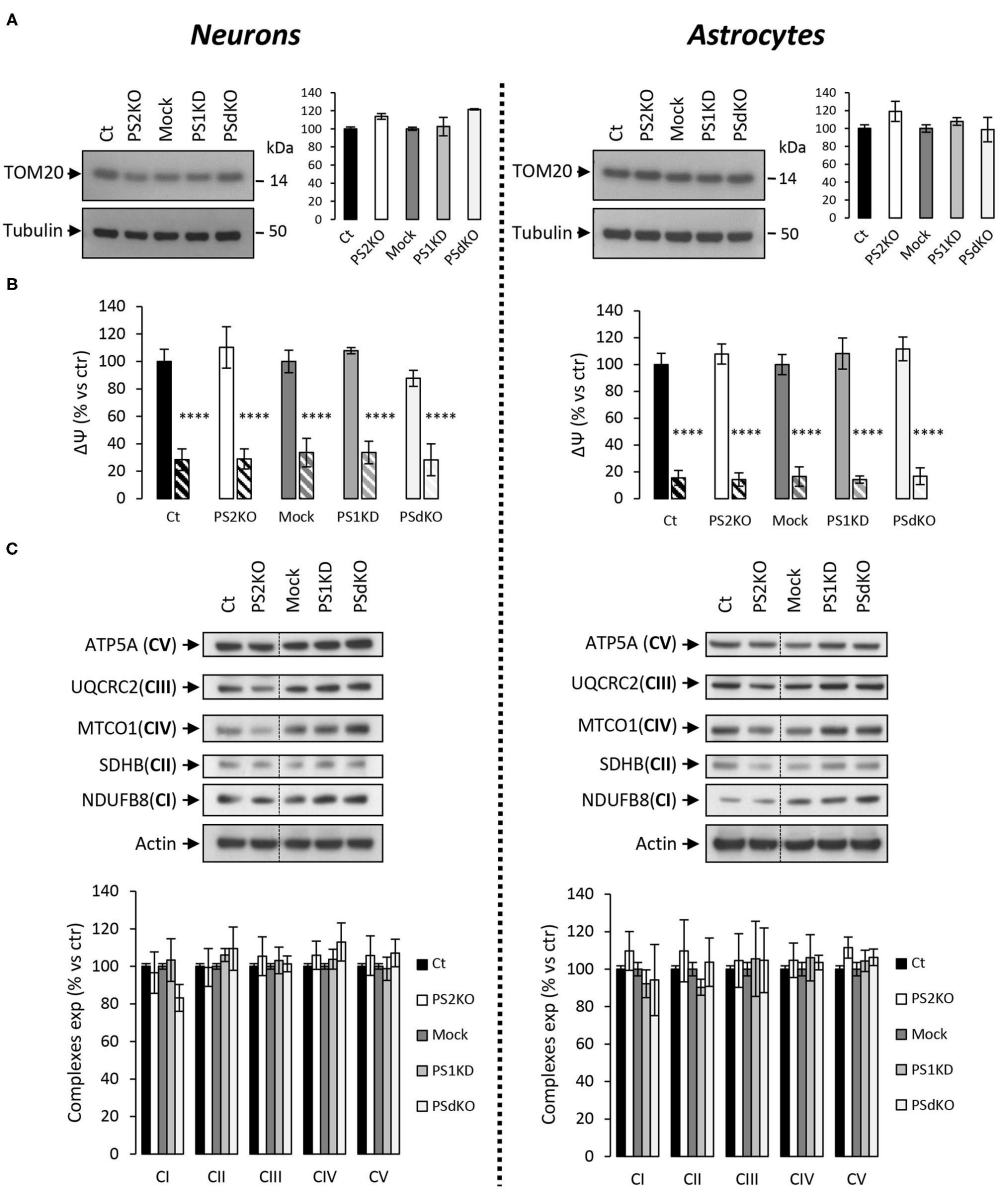
ΔΨ and OXPHOS complexes expression in PS-deficient primary nerve cells. Experiments were performed on primary DIV11 neuronal cultures and primary DIV24 astrocytes cultures. Cell conditions were Ct vs. PS2KO, Mock (infection control) vs. PS1KD and PSdKO. **(A)** Representative WBs and quantifications of TOM20 profile expression; in primary neuronal cell lysates (left panel) and in primary astrocyte cell lysates (right panel). Tubulin was used as a loading control. Results (mean ± sem) are expressed as percentage of the respective control (ctr = Ct or Mock) (min *N* = 2). Kruskal–Wallis test and Dunn's multiple comparison test. **(B)** ΔΨ was evaluated with the TMRM probe in the presence or absence of FCCP, an uncoupling agent used as control (striped columns). Fluorescence signal was measured with a plate reader and results are expressed as the percentage of the relative mean fluorescence of the respective control cells (ctr = Ct or Mock) (min *N* = 3). ANOVA and Tukey's multiple comparison test. ****p < 0.0001. **(C)** The expression level of representative protein subunits from each of the five mitochondrial complexes (NDUFB8 for CI; SDHB for CII; UQCRC2 for CIII; MTCO1 for CIV; ATP5A for CV) was evaluated by WB on cell lysates. Actin was used as a loading control. Dashed lines indicate that proteins were run on the same gel, but lanes are not contiguous. Quantifications of the different WBs (means ± sem) are given as percentage of signal measured in the respective control cells (Ct or Mock) (min *N* = 3). ANOVA and Tukey's multiple comparison test.

As a primary indicator of mitochondrial mass (Whitaker-Menezes et al., 2011), the expression level of the mitochondrial import receptor subunit TOM20 (Figure 3A) was first evaluated in cell lysates from primary neurons and astrocytes. No changes were observed, suggesting that deletion of PSs did not affect the mitochondrial mass in the cells tested. Mitochondrial membrane potential (ΔΨ) is crucial for energy production and it is the driving force generated by the electron transport chain (ETC) for ATP synthesis. ΔΨ is known to stay stable since its decrease is a strong indication signal of cell death (Uechi et al., 2006). We evaluated the ΔΨ with the TMRM probe (Figure 3B), using FCCP (an uncoupling agent abolishing ΔΨ) as a positive control. No significant differences in ΔΨ were observed between controls (Ct and Mock) and PS2KO, PS1KD, and PSdKO cells, neither in neurons nor in astrocytes. Since ATP synthase might work in reverse to keep ΔΨ stable (Uechi et al., 2006), a defect in ETC could still occur without being readily detectable by mitochondrial membrane potential measurements. We checked the expression of the ETC subunits by WB using a cocktail of antibodies targeting representative subunits of the five mitochondrial complexes (Figure 3C). We found no differences in the expression of any ETC subunit in PS1KD, PS2KO, or PSdKO neurons or astrocytes. This was rather unexpected since we previously reported a defect in oxidative phosphorylation (OXPHOS) capacity along with expression changes of the ETC subunits in PS2KO MEF cell lines (Contino et al., 2017).

### Mitochondrial Oxidative Phosphorylation and Bioenergetics Are Not Affected by the Absence of PS1 and/or PS2 in Primary Neurons and Astrocytes

**Figure 4:**
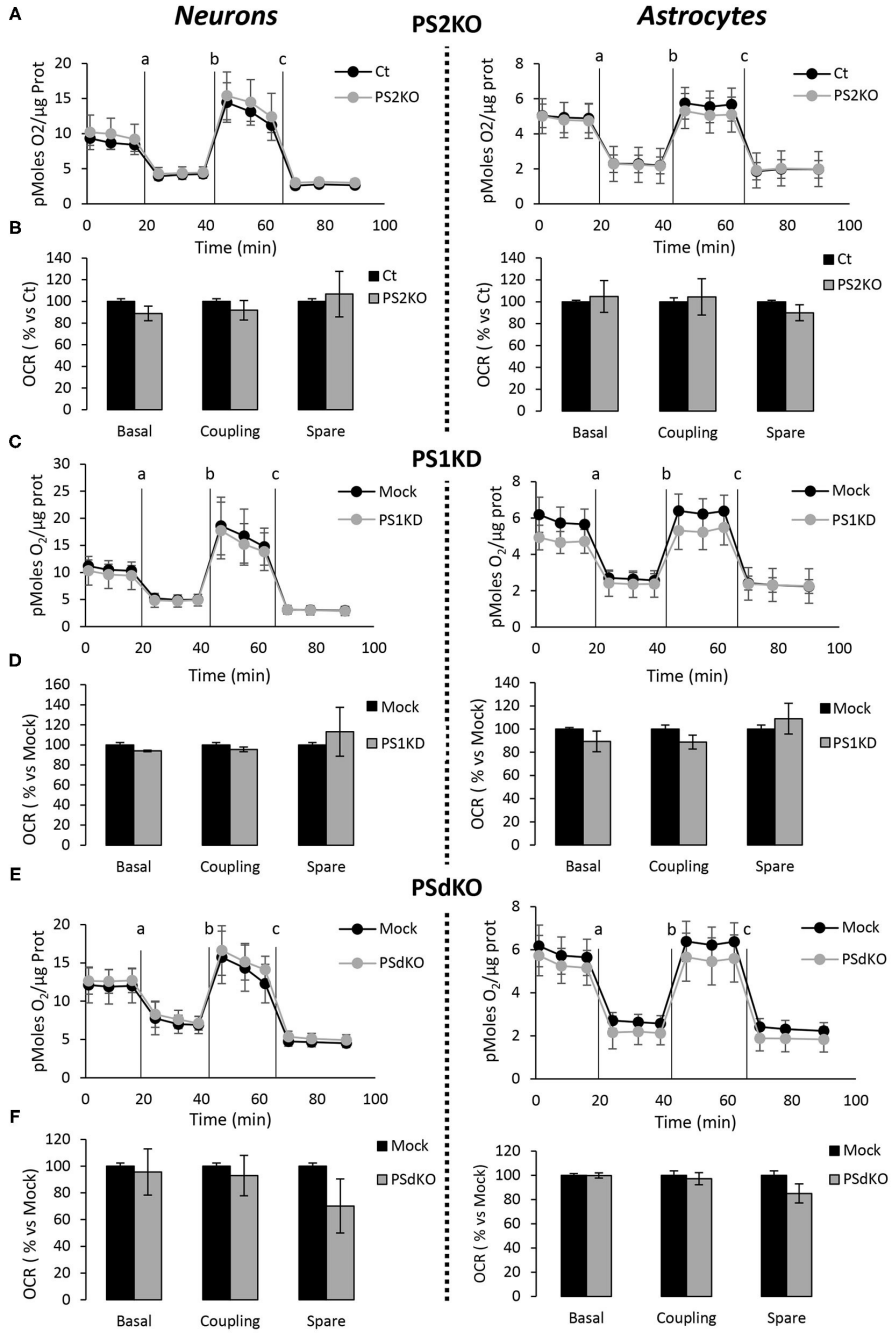
Assessment of the OXPHOS capacity in primary nerve cultures genetically deleted for PSs. Oxygen consumption rate (OCR) was evaluated by using the Seahorse XF96 bioenergetic analyzer. Experiments were carried out in primary DIV11 neuronal cultures (left panel) and in primary DIV24 astrocyte cultures (right panel). Cell conditions were wild-type non-infected (Ct) vs. PS2KO; control infection (Mock) vs. PS1KD and PSdKO. **(A,C,E)** General profile of the OCR with vertical lines indicating the time point at which the different compounds have been added: a. Oligomycin (CV inhibitor) b. FCCP (ΔΨ uncoupler) c. Rotenone (CI inhibitor) and antimycin A (CIII inhibitor). Values (means ± sem) are given in pmol O_2_/min/μg protein (min *N* = 3). **(B,D,F)** The basal respiration, the coupling ratio and the spare respiratory capacity were calculated according to the Cell Mito Stress Test kit's recommended protocol. Values (means ± sem) are given as percentage of signal measured in the respective control cells (Ct or Mock) (min *N* = 3). ANOVA and Tukey's multiple comparison test.

The absence of changes in ETC complexes levels is a biochemical indication that does not rule out the hypothesis that OXPHOS could be impaired in PS-deficient primary neurons or astrocytes. Indeed, the cocktail of OXPHOS antibodies used in our study targets only one subunit of each of the massive ETC complex. We evaluated the activity of the ETC by measuring the overall profile of oxygen consumption rate (OCR) and several related parameters (Figures 4A–C). The parameters measured were basal respiration, coupling (oxygen consumption devoted to ATP synthesis under resting conditions), and spare respiratory capacity (maximal uncoupled rate of respiration minus the basal rate). The absence of PS2 affected all these parameters in MEF PS2KO cell lines (Supplementary Figure 2). Strikingly, the general OCR measured was similar in the presence (Ct, Mock) or absence of PSs (PS1KD, PS2KO, PSdKO) in primary neurons and astrocytes (Figures 4A–C). To note, the OCR at basal state is running near the maximal respiratory capacity in astrocyte cultures. This could suggest that the cells are stressed. However, this kind of OCR profile is commonly observed in primary astrocyte cultures (Damiano et al., 2014; Logan et al., 2018; Neal et al., 2018). The GFAP staining (Figure 2) indicates that astrocytes in culture are indeed activated astrocytes (Liddelow and Barres, 2017; Dubovy et al., 2018). This activated astrocyte profile could explain the similar OCR values measured in astrocytes at basal state and upon stimulation with FCCP.

**Figure 5:**
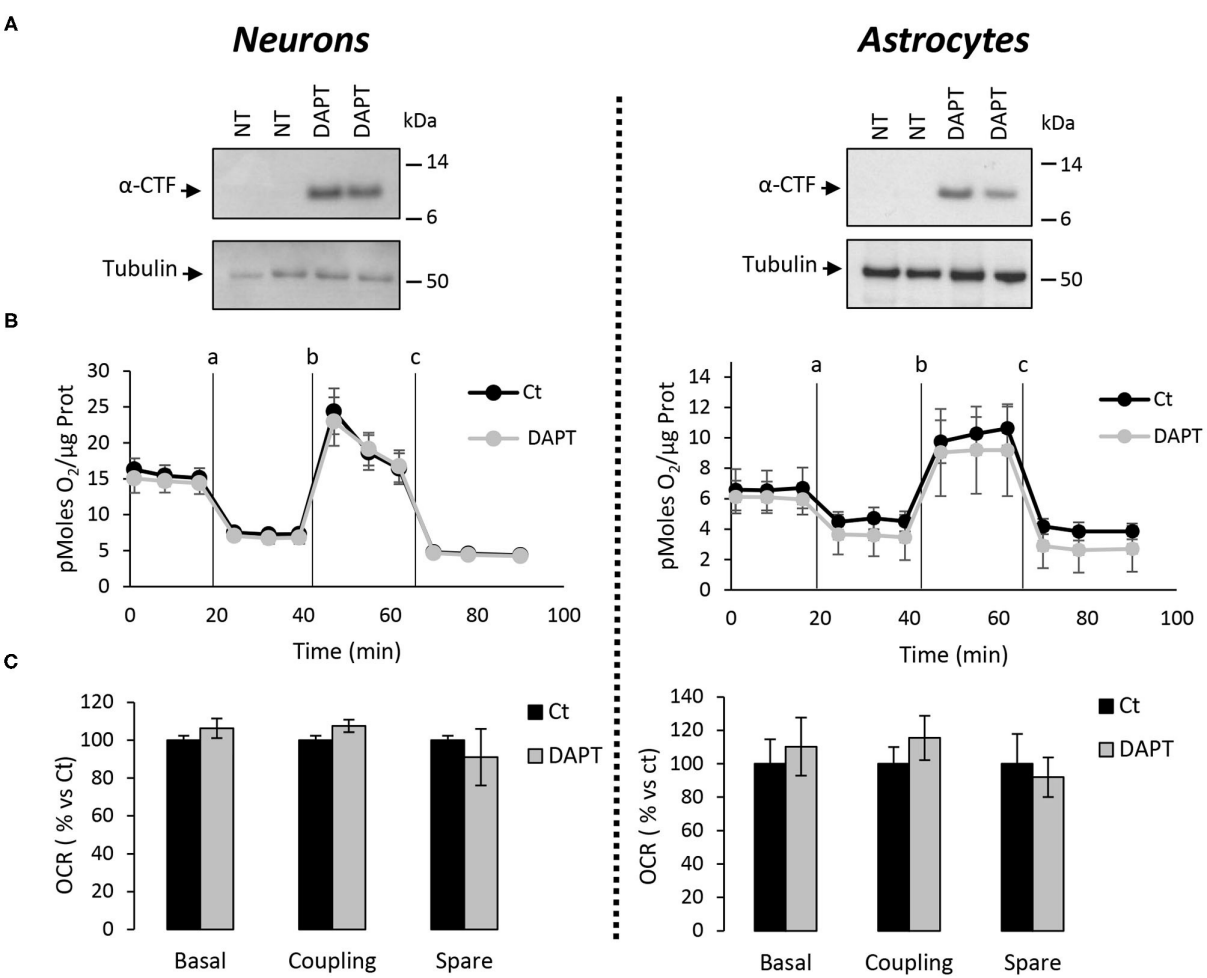
Assessment of the OXPHOS capacity in primary nerve cultures treated with DAPT. OCR was evaluated by using the Seahorse XF96 bioenergetic analyzer. Experiments were performed in primary neurons (DIV11) or primary astrocytes (DIV7) wild-type non-treated (NT or Ct) or treated with DAPT. **(A)** Accumulation of α-CTF upon treatment with 10 μM of DAPT for 24 h of control primary nerve cultures was evaluated by WB with an antibody targeting the C-terminal region of APP. Tubulin was used as loading control. **(B)** General profile of the OCR with vertical lines indicate the time point at which the different compounds have been added: a. Oligomycin (CV inhibitor) b. FCCP (ΔΨ uncoupler) c. Rotenone (CI inhibitor) and antimycin A (CIII inhibitor). Values (means ± sem) are given in pmol O_2_/min/μg protein (*N* = 3). **(C)** The basal respiration, the coupling ratio, and the spare respiratory capacity were calculated according to the Cell Mito Stress Test kit's recommended protocol. Values (means ± sem) are given as percentage of signal measured in control cells (Ct). Student's *t*-test for neurons results (*N* = 3) and Mann-Whitney test for astrocytes results (*N* = 2).

**Figure 6:**
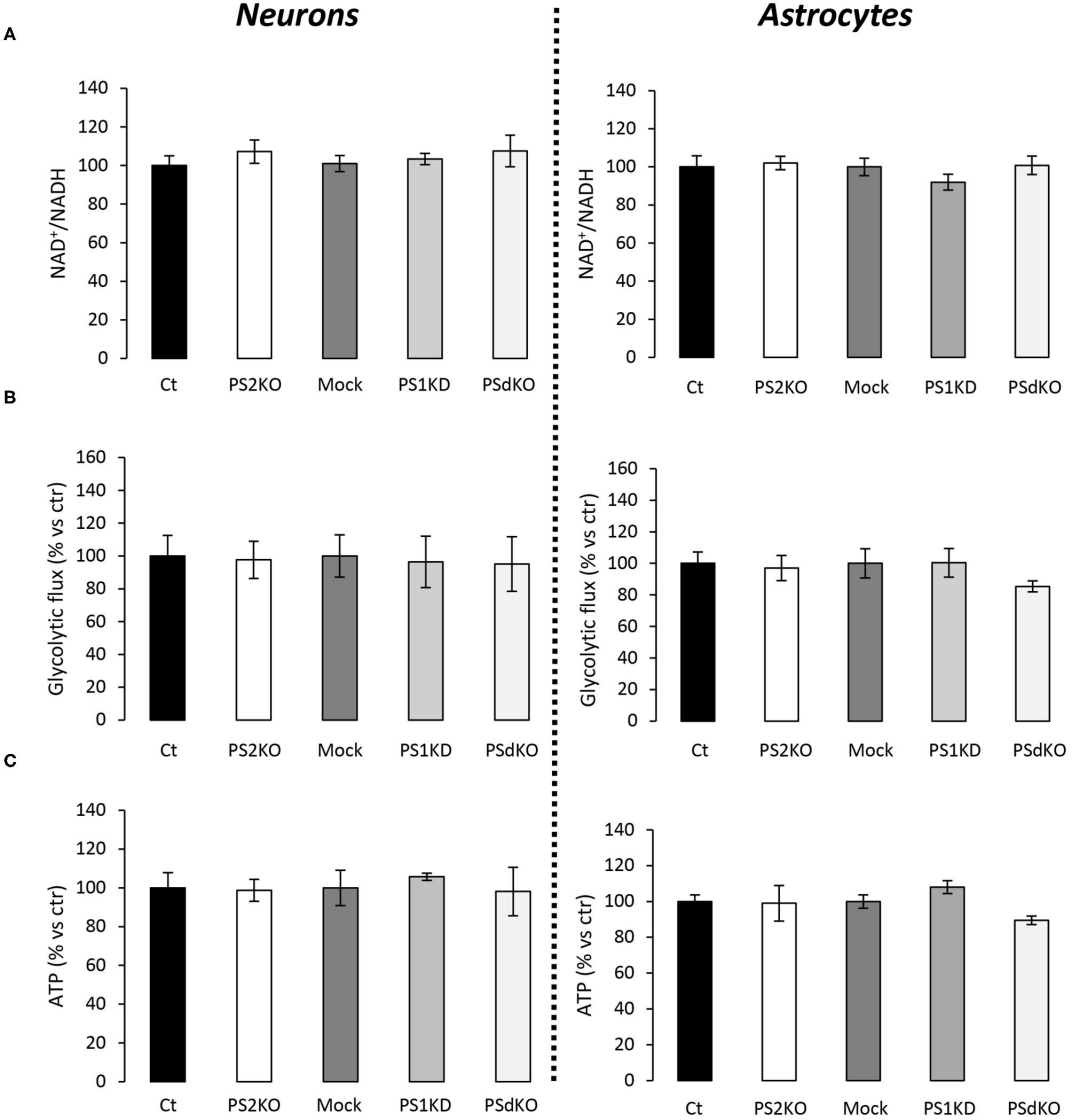
Evaluation of ATP levels, NAD^+^/NADH ratio, and glycolytic flux in primary nerve cultures. Experiments were performed respectively at DIV11 and DIV24 in neuronal (left panel) and astrocytes (right panel) cultures. Cell conditions were wild-type non-infected (Ct) vs. PS2KO; Mock (infection control) vs. PS1KD and PSdKO. (ctr = Ct or Mock). **(A)** NAD^+^/NADH ratio was quantified by a bioluminescent kit (min *N* = 3). **(B)** Glycolysis rate was determined by the detritiation rate of [3-^3^H] glucose after a 30 min incubation. Data were normalized to protein content (min *N* = 3). **(C)** ATP level was quantified by a bioluminescent kit (min *N* = 3). ANOVA and Tukey's multiple comparison test.

**Figure 7:**
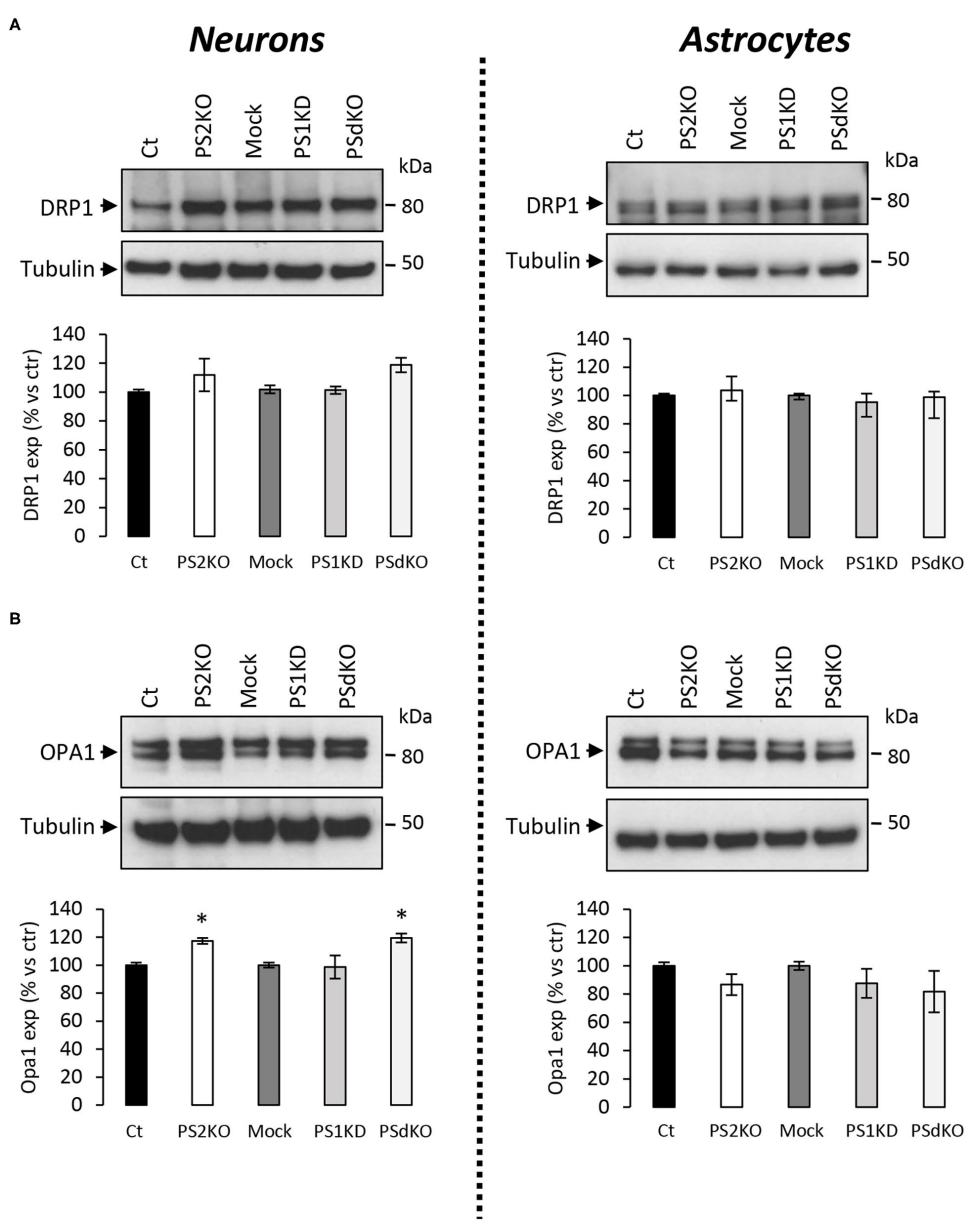
Expression of mitochondrial fusion and fission proteins in PS-deficient primary nerve cells. Experiments were performed on primary DIV11 neuronal cultures and primary DIV24 astrocytes cultures. Cell conditions were Ct vs. PS2KO, Mock (infection control) vs. PS1KD and PSdKO. **(A)** Representative WBs and quantifications of DRP1 profile expression; in primary neuronal cell lysates (left panel) and in primary astrocyte cell lysates (right panel). Tubulin was used as a loading control. Results (mean ± sem) are expressed as percentage of the respective control (ctr = Ct or Mock) (min *N* = 2). Kruskal–Wallis test and Dunn's multiple comparison test. **(B)** Representative WBs and quantifications of OPA1 profile expression; in primary neuronal cell lysates (left panel) and in primary astrocyte cell lysates (right panel). Tubulin was used as a loading control. Results (mean ± sem) are expressed as percentage of the respective control (ctr = Ct or Mock) (min *N* = 4). ANOVA and Tukey's multiple comparison test, *p < 0.05.

The major outcome of genetic deletion of PSs is to down-regulate or abolish γ-secretase activity in cells. Another way to address the role of γ-secretase activity in OXPHOS is to block γ-secretase activity with pharmacological inhibitors. To that end, we treated primary neurons and astrocytes for 24 h with 10 μM of DAPT, a concentration that efficiently blocks γ-secretase activity (Figure 5A). We measured the OCR (Figures 5B,C) and observed no changes in DAPT-treated cells when compared to non-treated cells. This confirms that γ-secretase activity is not involved in OXPHOS, in agreement with the results obtained in PS-deficient neurons and astrocytes. We next measured the NAD^+^/NADH ratio (Figure 6A) and glycolytic flux (Figure 6B), parameters related to bioenergetics. NADH is an electron donor used by the first complex of the ETC. Glycolysis can either produce intermediates for OXPHOS or produce ATP and lactate depending on the oxidative status. These parameters were found to be altered in PS2KO MEF cell lines (Contino et al., 2017). We did not observe any changes in these indicators in primary neurons or astrocytes in the absence of PSs. In agreement, ATP levels were stable in all cell types (Figure 6C). All these data strongly support that the mitochondrial activity and related bioenergetics are not dependent on PSs in neurons and astrocytes, on the contrary to what was observed in MEF (Contino et al., 2017). Finally, we checked for possible mitochondrial fusion/fission defects in PS-deficient cells. Mitochondria are very dynamic entities and their shape and length can change erratically due to fusion and fission processes under certain circumstances such as cell division or especially in response to stress. We evaluated by WB two key proteins of mitochondrial fusion (OPA1) and fission (DRP1) as indicators of altered mitochondrial dynamics (Figures 7A,B). We found only a very small but significant increase in OPA1 in PS2KO and PSdKO neurons that is not mirrored by the DRP1 profile. Thus, subtle changes in mitochondrial dynamics could occur in PS2-deficient cells without affecting mitochondrial OXPHOS and related bioenergetics (Figures 4, 5).

**Figure 8:**
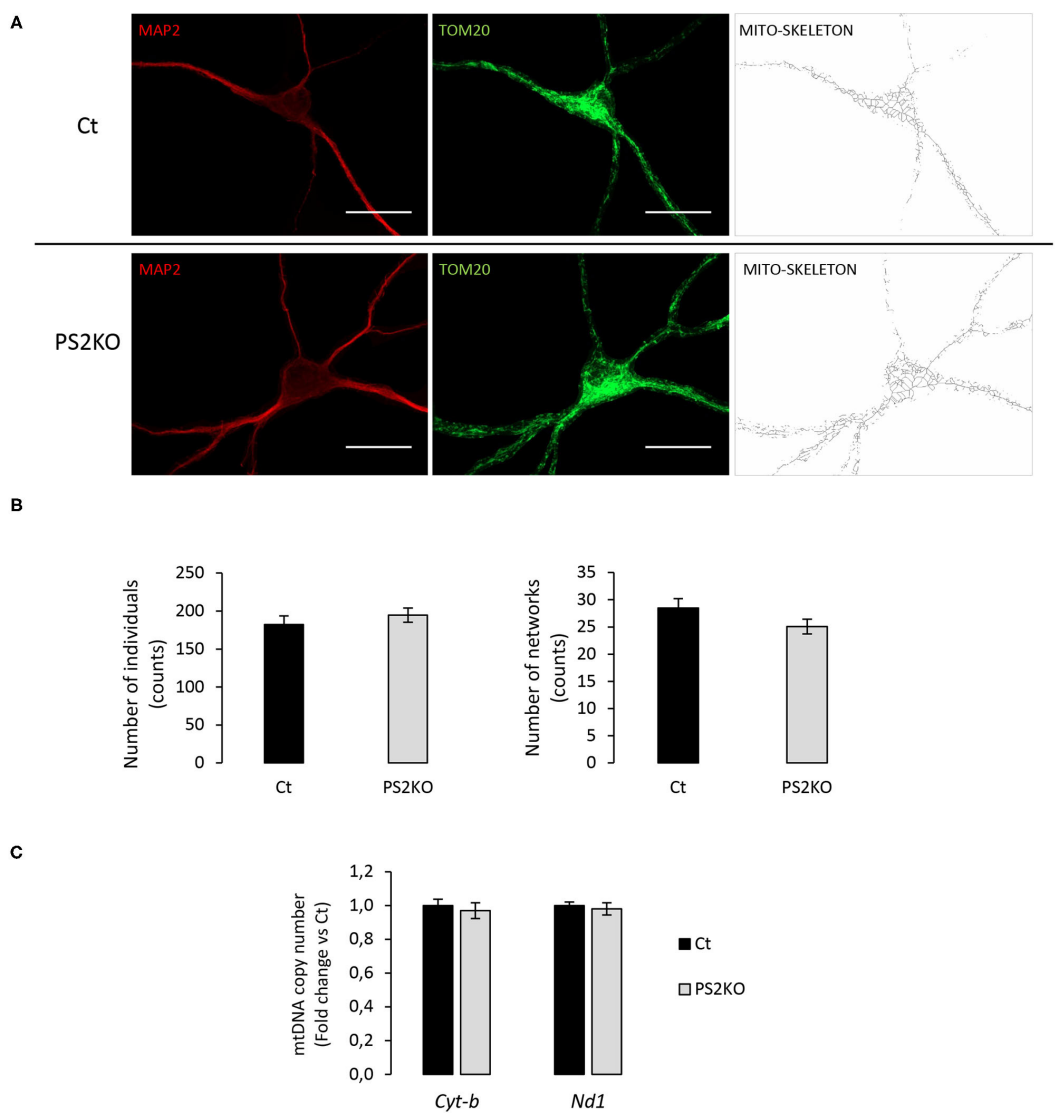
Evaluation of mitochondrial morphology and content in PS2KO neurons. **(A)** Mitochondria from wild-type non-infected (Ct) and PS2KO neurons at DIV11 were stained with an anti-TOM20 antibody (green) and mature neurons were identified with MAP2 (red). Confocal microscope images were acquired and TOM20 images were processed using the MiNA toolset to generate accurate mitochondrial skeletons for further analysis. Scale bar = 20 μm. **(B)** Quantification of individuals (counts) and networks (counts) are shown in the graphs. Results are presented as mean ± sem. Student's *t*-test (*n* = 55 from *N* = 3 independent experiments). **(C)** RT-qPCR analysis of mitochondrial DNA content (*Cyt-b* and *Nd1* mitochondrial genes) in wild-type non-infected (Ct) and PS2KO neurons at DIV11. Results (mean ± sem) are expressed as percentage of the respective control (Ct) (min *N* = 4).

To further investigate the cellular effects of such protein changes in PS2KO neurons, and since previous results from our group demonstrated specific alterations in mitochondrial function and content in immortalized PS2KO MEF cells (Contino et al., 2017), we decided to evaluate mitochondrial morphology in WT (Ct) and PS2KO neurons by immunofluorescent staining against TOM20 (Figure 8A). A semi-automated and detailed characterization was accomplished by using the Mitochondrial Network Analysis (MiNA) toolset in ImageJ, which allows the obtention of parameters to quantitatively capture the morphology of the mitochondrial network (Valente et al., 2017). Briefly, confocal images of mature neurons (positive for MAP2) were processed to enhance their resolution and converted to binary images, finally producing the mitochondrial skeleton morphology. In all analyzed cells mitochondria were interconnected, thus forming an intracellular network, and no differences were observed when evaluating the mean number of mitochondria (individuals: unbranched, punctate organelles) or networks (branched, reticular structure of fused mitochondria) (Figure 8B). Additionally, no differences were observed in the mean number of junctions, end-points, and slab voxels (data not shown), suggesting an unaltered shape and distribution of the mitochondrial network together with the same degree of network complexity between genotypes. Finally, in order to confirm the lack of mitochondrial alterations in PS2KO neurons, a RT-qPCR-based mtDNA content analysis was performed in those cells, indicating normal mitochondrial mass in response to PS2 depletion (Figure 8C).

### Metabolic Characterization of PS2 Deficient Primary MEF

**Figure 9:**
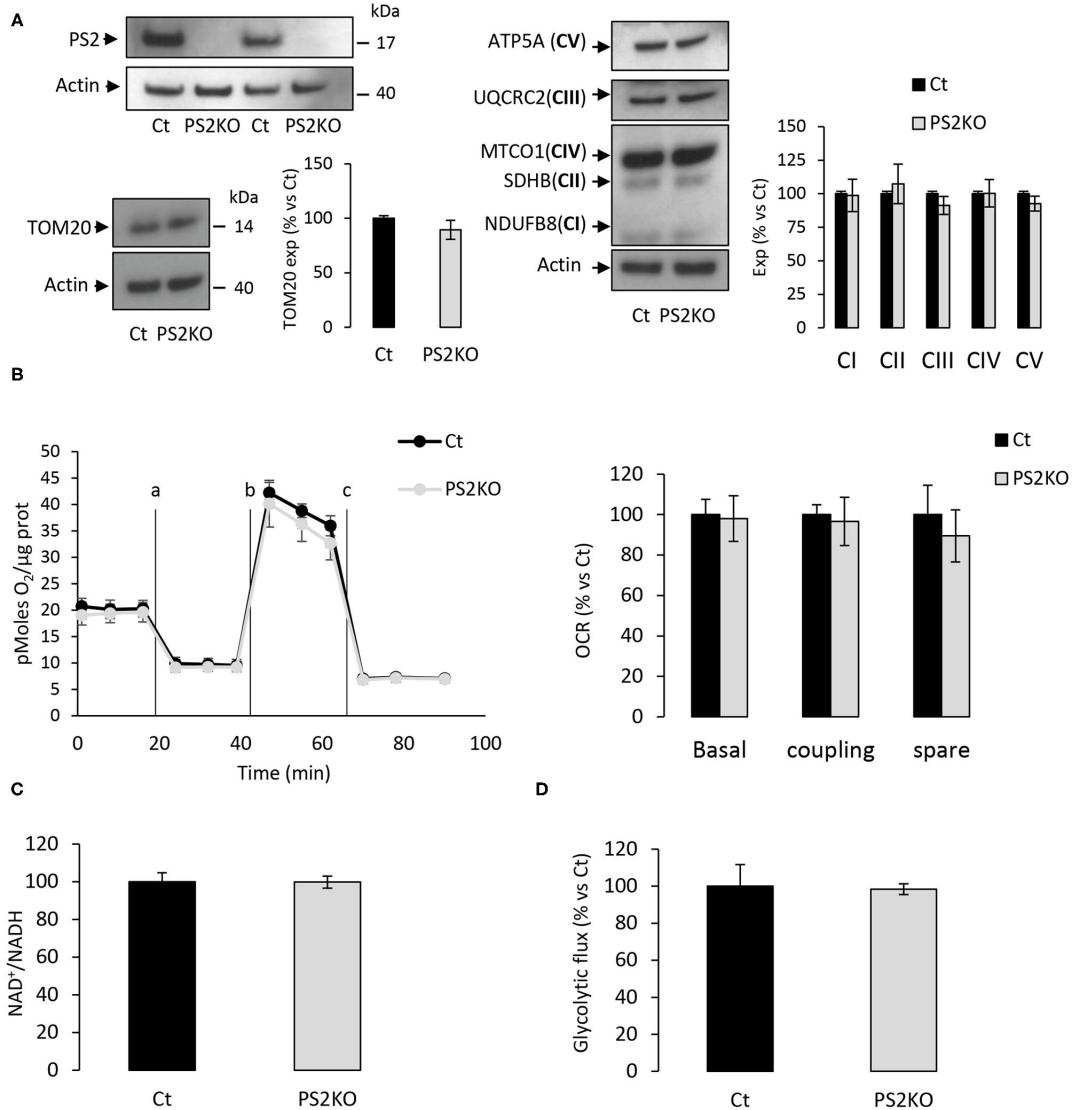
Metabolic characterization of PS2 deficient primary MEF. Experiments were carried out in primary MEF derived from E16 WT mice (Ct) or PS2KO mice (PS2KO). **(A)** Expression profile of PS2 (left panel, up), TOM20 (left panel, down), and representative protein subunits from each of the five mitochondrial complexes (right panel; NDUFB8 for CI; SDHB for CII; UQCRC2 for CIII; MTCO1 for CIV; ATP5A for CV), evaluated by WB on cell lysates. Actin was used as a loading control. Quantifications of the different WBs (means ± sem) are given as percentage of signal measured in control cells (Ct) (min *N* = 3). Student's *t*-test. **(B)** Left panel: General OCR profile of primary MEF Ct vs. PS2KO. Values (means ± sem) are given in pmol O_2_/min/μg protein. Right panel: The basal respiration, the coupling ratio and the spare respiratory capacity. Values (means ± sem) are given as percentage of signal measured in control cells (Ct) (*N* = 3). **(C)** NAD^+^/NADH ratio was quantified by a bioluminescent kit (*N* = 4). **(D)** Glycolytic rate was determined by the detritiation rate of [3-^3^H] glucose after a 30 min incubation. Data were normalized to protein content (*N* = 3). Student's *t*-test.

Since no metabolic defect was observed in primary neuronal and astrocyte cells, we hypothesized that the phenotype previously observed in MEF cells could be exclusively peripheral. Indeed, the general OCR profile and related parameters were defective in PS2KO MEF cell lines and restored after stable re-expression of human PS2 (Supplementary Figure 2). To further investigate this idea, we decided to generate primary MEF derived from E16 WT (Ct) or PS2KO mice. We measured the expression of TOM20 and subunits of OXPHOS complexes (Figure 9A) and did not observe any differences between Ct and PS2KO fibroblasts. The activity of the ETC was also stable in the absence of PS2 as shown with the general OCR profile and the related parameters (Figure 9B). Finally, NAD^+^/NADH ratio and glycolytic flux (Figures 9C,D) were not modified either, indicating a metabolic stability in primary fibroblasts lacking PS2. The metabolic phenotype observed in primary fibroblasts is not consistent with the one reported in immortalized MEF, and could reflect deep differences between primary and immortalized cells.

## Discussion

PSs play a major role in cell physiology and AD pathology as catalytic subunits of the γ-secretase complex. The γ-secretase is a multiprotein membrane complex, involved in regulated intramembrane proteolysis (RIP). Up to 90 membrane proteins have been identified as substrates of the γ-secretase (Haapasalo and Kovacs, 2011). The major substrates are Notch and the Amyloid Precursor Protein (APP), which respectively play a critical role during development and in the amyloid pathology found in AD (Hardy, 2006). PSs have also been implicated in other cellular functions, including calcium homeostasis, cell-cell adhesion, membrane trafficking, and Wnt signaling (Otto et al., 2016). The most reported non-catalytic functions of PSs are related to calcium homeostasis (Nelson et al., 2007; Cheung et al., 2008; Wu et al., 2013). PSs contribute to the building of functional ER/mitochondria interfaces called mitochondria-associated membranes (MAMs) (Area-Gomez et al., 2009; Brunello et al., 2009; Filadi et al., 2016). MAMs control calcium shuttling between ER and mitochondria, interconnecting calcium homeostasis and mitochondrial function. We previously observed in MEF cell lines that the absence of PS2 led to a decrease in OXPHOS activity and in the expression of ETC proteins, along with an increased anaerobic glycolysis that sustains the ATP production. The effects observed were cell-autonomous since defects in bioenergetics were rescued by the stable expression of human PS2 in PS2KO cells (Contino et al., 2017). These indications supported the role of presenilins, and more precisely PS2 in mitochondrial function, in agreement with other studies carried out in the same model (MEF) or in neuronal cell lines. PS2 but not PS1 deficiency was reported to alter mitochondrial respiration (Behbahani et al., 2006). PS2 was found to modulate calcium shuttling between the ER and mitochondria, a critical process for OXPHOS stimulation and thereby mitochondrial activity (Zampese et al., 2011). In agreement, a study reported that PSs are enriched in MAMs (Area-Gomez et al., 2009).

We further investigated in this study the role of PSs in mitochondrial function by using mouse primary neurons and astrocytes. Contrary to what was previously observed, we found that the absence of PS1 or PS2 and even of both (PSdKO) did not affect mitochondrial ETC and related bioenergetic parameters in primary nerve cells. Moreover, the morphology of the mitochondrial network and the quantity of mtDNA were not changed either in PS2KO neurons. Importantly, we also did not observe any change in primary PS2-deficient fibroblasts, in opposition to the results obtained in immortalized fibroblasts (MEF cell lines). This raises important points about the interpretation of the role of PSs in mitochondrial functions across cellular models. First, the PS2KO MEF cell lines, which have been widely used so far, can genetically derive and acquire clonal properties, with the inherent risk of artifacts or misinterpretation of the data (Kaur and Dufour, 2012). Immortalized cell lines can acquire mutations with subcultures and these mutations can interfere with the cellular phenotype. However, the rescue experiments that we performed in a previous study indicated that the mitochondrial defects observed in PS2KO MEF cell lines are truly PS2-dependent (Contino et al., 2017). Second, the immortalization process might be responsible for the PS2-dependent phenotype observed in MEF. The fact that a PS2-dependent phenotype is observed only in immortalized cells can be related to data from the literature. PSs are involved in different cellular pathways related to cancer, like Notch and Wnt pathways (Xia et al., 2001; Andersson and Lendahl, 2014; Li et al., 2016). An increase in lung tumor formation through peroxiredoxin 6 (PRDX6) activation was also reported in PS2KO mice. The proposed underlying mechanism involves the PS2-dependent γ-secretase cleavage of PRDX6 that inhibits its critical activity in cell growth (Yun et al., 2014; Park et al., 2017). Another study showed that PSs are involved in epidermal growth and transformation by regulating the epidermal growth factor receptor (EGFR) signaling (Rocher-Ros et al., 2010). It is also important to take into account that the immortalization process used for the generation of MEF cell lines relied on SV40 Antigen T expression (De Strooper et al., 1999), which shows similarities with tumor development due to the large T antigen forming complexes with pRB-1 and p53 (Hubbard and Ozer, 1999; Pipas, 2009). In agreement, the inhibition of γ-secretase activity is suggested as a potential approach for cancer treatment. Interestingly, PSs have been associated to regulation of proteins such as Akt, HIFα, or β-catenin (Xia et al., 2001; Kang et al., 2005; De Gasperi et al., 2010). Those proteins contribute to the Warburg effect, which states that most cancer cells produce lactic acid from glucose even under non-hypoxic conditions and despite functional mitochondria (Koppenol et al., 2011). Many pieces of evidence relate thus PSs function to cancer processes, not only to neurodegenerative diseases. Our data clearly indicate that the absence of PS2 has a different outcome when measured in primary fibroblast or in immortalized fibroblast. The large amount of data reported so far in MEF cell lines (Behbahani et al., 2006; Tu et al., 2006; Brunello et al., 2009; Filadi et al., 2016; Pera et al., 2017) might be relevant to describe the role of PSs and γ-secretase activity in cancer models, which are difficult to transpose to a neurodegenerative context.

To that end, we addressed the role of PSs in mitochondrial function in primary neurons and astrocytes. Neurons are a prime target of neurodegeneration, and a major function of astrocytes is to support neuronal activity. Impairment of the neuronal network and activity underlies the gradual memory and other cognitive deficits in AD (Long and Holtzman, 2019). Although neurons are the most studied nerve cell type, astrocytes also play a key role in AD pathogenesis (Verkhratsky et al., 2010). Astrocytes and neurons are known to be metabolically different. Neurons rely primarily on mitochondrial oxidative phosphorylation for energetic supply and astrocytes on glycolysis. We did not observe any metabolic defects in our PS-deficient primary neuronal cultures. Indeed, the OXPHOS system (activity and expression) as well as the ΔΨ, glycolysis, and NAD^+^/NADH ratio were comparable between control, PS2KO, PS1KD, and PSdKO cells. Since astrocytes are more glycolytic than neurons (Kasischke et al., 2004; Castelli et al., 2019), we hypothesized that astrocytes would be close, in terms of metabolic phenotype, to the MEF cell line we previously analyzed (Contino et al., 2017). However, all the metabolic parameters evaluated in primary astrocytes were not altered by the absence of PSs. This leads us to conclude that the lack of PSs does not affect neither the OXPHOS, nor related metabolic aspects, such as glycolysis and NAD^+^/NADH ratio in primary nerve cells. In support of these observations, the analysis of the morphology and complexity of the mitochondrial network, as well as its mass, does not show differences between control and PS-deficient neurons. This result is coherent with all the data obtained regarding mitochondrial function; indeed, the preservation of a correct and interconnected network of mitochondria is crucial to cell function (Chen and Chan, 2004; Lackner, 2014). To note, differences between nerve cells and peripheral cells have been reported in a metabolic study comparing primary neurons, astrocytes, and fibroblasts cultures deficient for the mitochondrial complex I subunit NDUFS4, a model for the mitochondrial Leigh syndrome, a severe neurological disease (Bird et al., 2014). The ΔΨ and ATP synthesis were impaired in the NDUFS4 KO primary MEF, with an increase in ROS generation and an altered sensitivity to cell death. In contrast, NDUFS4 KO primary neurons and astrocytes displayed only impaired ATP generation. This underlines the importance of the cellular model and experimental set-up when investigating alteration of mitochondrial function related to a neuronal pathology.

Still, the fact that the absence of PSs does not affect the basal mitochondrial-related bioenergetics in astrocytes and neurons is rather intriguing. Considering the expression profiles of the *PSEN1* and *PSEN2* genes throughout the body, one could expect to have distinct phenotypes related either to PS1 or PS2 deficiency. PS1 was suggested to be more important in CNS and PS2 in peripheral organs (Lee et al., 1996). The absence of mitochondrial defects in PSdKO is even more unexpected, considering the broad array of functions attributed to PSs (Zhang et al., 2013; Wolfe, 2019), which process more than 90 membrane proteins (Haapasalo and Kovacs, 2011; Wolfe, 2019). Cortical brain sections from conditional PSdKO mice show several pathological features such as neurodegeneration, astrogliosis, and even swollen mitochondria (Saura et al., 2004; Wines-Samuelson et al., 2010). The experimental set-up and environment should be taken into consideration to give a precise interpretation to data obtained across different models. All our experiments were performed on primary cells that were cultured in low glucose (5 mM) medium, to reflect a more physiological condition. Neurons and astrocytes were mature and differentiated (DIV11 for neurons and DIV24 for astrocytes), and tested at basal and resting state. Challenging the cells might be a key point for further investigations to unravel a deficit that could be masked in basal conditions. The time of the culture, especially for neurons, is also an important parameter. Performing the experiments on aged cultures would be of interest to check if mitochondrial functions evolves distinctly in PS-deficient cells upon aging. Cultures could be supplied with different types of cell fuels (pyruvate, fatty acid…), cells could be challenged with depolarization or hypoxia to perhaps unravel a PS-dependent phenotype in specific contexts. Indeed, the brain tissue is much more complex than *in vitro* cultures, in which disrupted PS-dependent cell interactions could account for changes in energy homeostasis (Saura et al., 2004; Wines-Samuelson et al., 2010).

In conclusion, our study provides evidence for the lack of mitochondrial alterations in PS1KD, PS2KO, and even PSdKO neurons and astrocytes, as well as in PS2KO primary MEF. This is in contradiction with previous observations in neuronal cell lines and immortalized fibroblasts. Thus, immortalized cells might provide relevant results regarding the role of PSs in mitochondrial activity and bioenergetics related to cancer processes, in which PSs involvement have already been reported. However, the contribution of PSs to alterations in mitochondrial activity related to neurodegenerative processes, such as AD, needs to be critically readdressed and further explored in brain models.

## Data Availability Statement

All datasets generated for this study are included in the article/Supplementary Material.

## Ethics Statement

The animal study was reviewed and approved by UCLouvain animal care committee's regulations (code number 2016/UCL/MD/016).

## Author Contributions

SC performed research, analyzed data, and wrote the paper. NS and CV performed research and analyzed data. DV performed research. VP, SS, and LB analyzed data. PK-C designed research and wrote the paper. All authors contributed to the article and approved the submitted version.

## Conflict of Interest

The authors declare that the research was conducted in the absence of any commercial or financial relationships that could be construed as a potential conflict of interest.

## Abbreviations

ΔΨ: mitochondrial membrane potential
2R2: PS2 knockout rescued PS2
CI-CV: complexes I-V
Ct: control
DTAB: dodecyltrimethylammonium bromide
ETC: electron transport chain
FCCP: carbonyl cyanide-4-(trifluoromethoxy) phenylhydrazone
GFAP: glial-fibrillary acidic protein
MAM: mitochondria-associated membranes
MAP-2: microtubule-associated protein 2
MEF: mouse embryonic fibroblasts
MiNA: mitochondrial network analysis
OCR: oxygen consumption rate
OXPHOS: oxidative phosphorylation
PS: presenilin
ROS: reactive oxygen species
TMRM: tetramethylrhodamine methyl ester.
