# Supplementary figures and images for: Presenilin-Deficient Neurons and Astrocytes Display Normal Mitochondrial Phenotypes
Source: Front Neurosci. 2021 Jan 22;14:586108. doi: 10.3389/fnins.2020.586108 (PMC7862347; doi:10.3389/fnins.2020.586108)

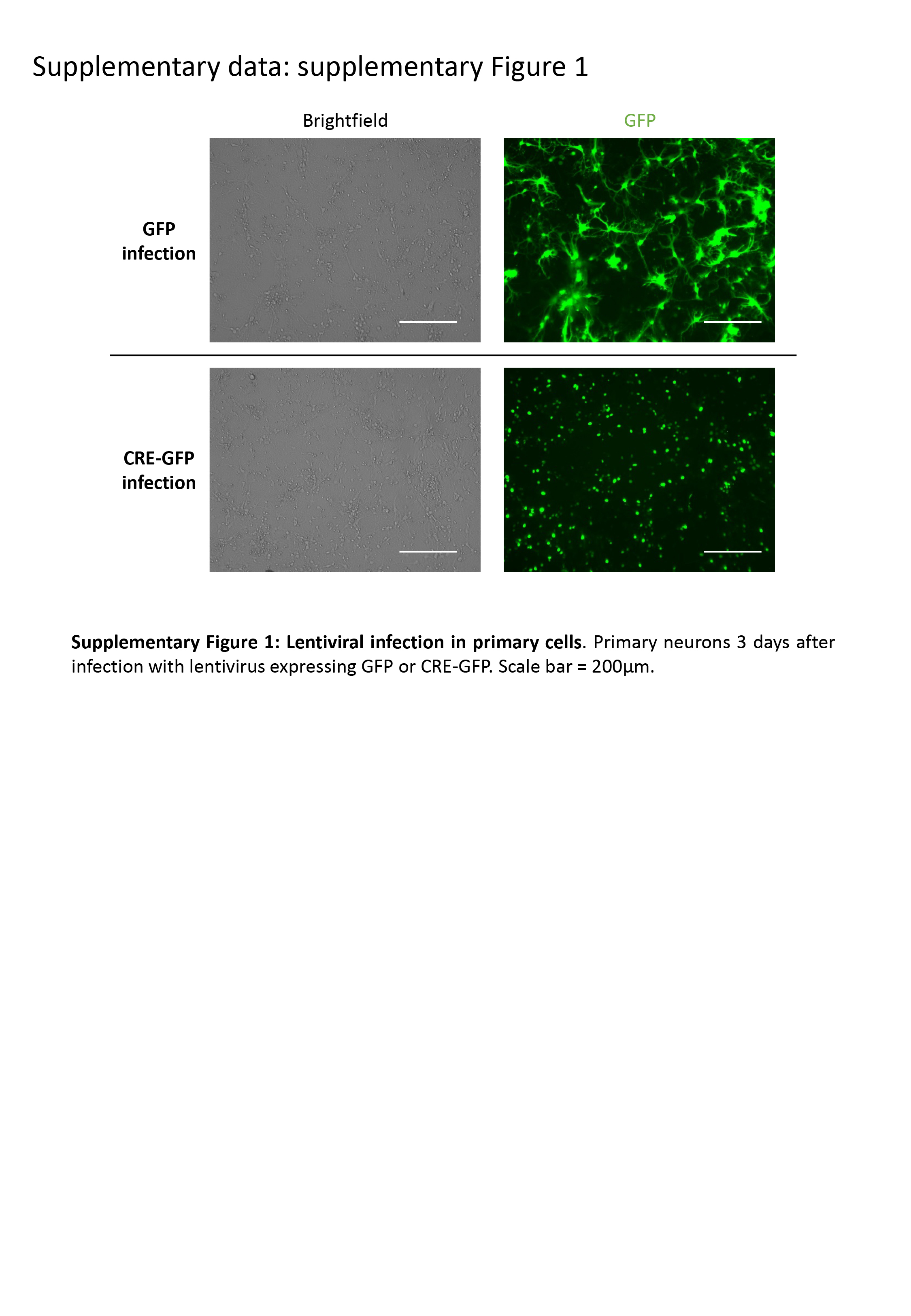

Supplement: Supplementary file 1 [file Image_1.JPEG]

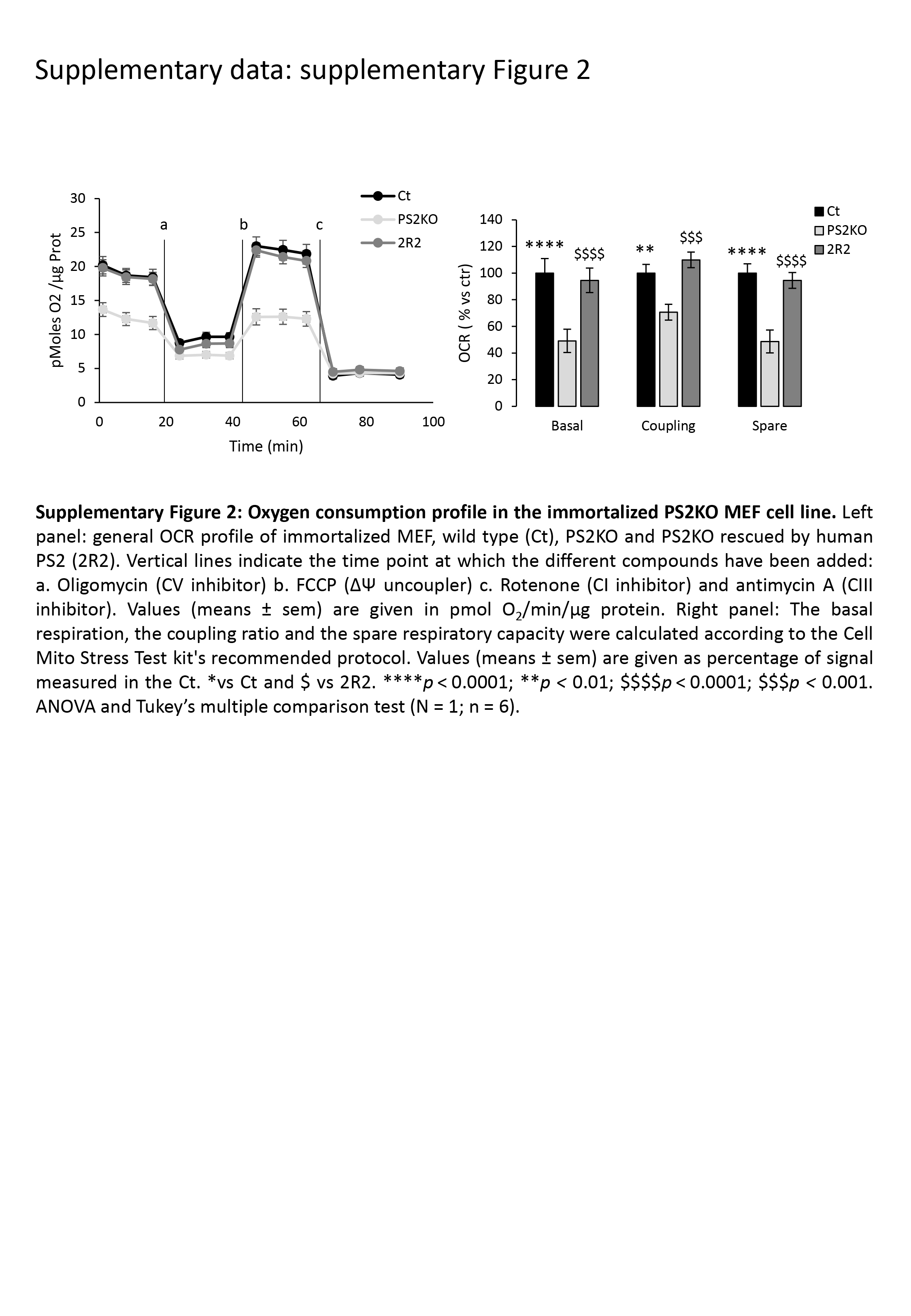

Supplement: Supplementary file 2 [file Image_2.JPEG]
